# Mechanical Comparison between Fenestrated Endograft and Physician-Made Fenestrations

**DOI:** 10.3390/jcm12154911

**Published:** 2023-07-26

**Authors:** Jérémie Jayet, Jennifer Canonge, Frédéric Heim, Marc Coggia, Nabil Chakfé, Raphaël Coscas

**Affiliations:** 1 Department of Vascular Surgery, Ambroise Paré University Hospital, Assistance Publique—Hôpitaux de Paris (AP-HP), 92100 Boulogne-Billancourt, Francemarc.coggia@aphp.fr (M.C.); raphael.coscas@aphp.fr (R.C.); 2 Groupe Européen de Recherche sur les Prothèses Appliquées à la Chirurgie Vasculaire (GEPROVAS), 67085 Strasbourg, France; frederic.heim@uha.fr (F.H.); nabil.chakfe@chru-strasbourg.fr (N.C.); 3 Laboratoire de Physique et Mécanique Textiles (LPMT), ENSISA, 68093 Mulhouse, France; 4 UMR 1018, Inserm-Paris11—CESP, Versailles Saint-Quentin-en-Yvelines University, Paris-Saclay University, Paul Brousse Hospital, 94800 Villejuif, France; 5 Department of Vascular Surgery, Henri Mondor University Hospital, 94010 Créteil, France; 6 Department of Vascular Surgery and Kidney Transplantation, Hôpitaux Universitaires de Strasbourg, Université de Strasbourg, 67084 Strasbourg, France

**Keywords:** aortic aneurysm, endovascular abdominal aortic aneurysm repair (EVAR), juxtarenal aortic aneurysm

## Abstract

Introduction: A fenestrated endograft (FE) is the first-line endovascular option for juxta and pararenal abdominal aortic aneurysms. A physician-modified stent-graft (PMSG) and laser in situ fenestration (LISF) have emerged to circumvent manufacturing delays, anatomic standards, and the procedure’s cost raised by FE. The objective was to compare different fenestrations from a mechanical point of view. Methods: In total, five Zenith Cook fenestrations (Cook Medical, Bloomington, IN, USA) and five Anaconda fenestrations (Terumo Company, Inchinnan, Scotland, UK) were included in this study. Laser ISF and PMSG were created on a Cook TX2 polyethylene terephthalate (PET) cover material (Cook Medical, Bloomington, IN, USA). In total, five LISFs and fifty-five PMSG were created. All fenestrations included reached an 8 mm diameter. Radial extension tests were then performed to identify differences in the mechanical behavior between the fenestration designs. The branch pull-out force was measured to test the stability of assembling with a calibrated 8 mm branch. Fatigue tests were performed on the devices to assess the long-term outcomes of the endograft with an oversized 9 mm branch. Results: The results revealed that at over 2 mm of oversizing, the highest average radial strength was 33.4 ± 6.9 N for the Zenith Cook fenestration. The radial strength was higher with the custom-made fenestrations, including both Zenith Cook and Anaconda fenestrations (9.5 ± 4.7 N and 4.49 ± 0.28 N). The comparison between LISF and double loop PMSG highlighted a higher strength value compared with LISF (3.96 N ± 1.86 vs. 2.7 N ± 0.82; *p*= 0.018). The diameter of the fenestrations varied between 8 and 9 mm. As the pin caliber inserted in the fenestration was 9 mm, one could consider that all fenestrations underwent an “elastic recoil” after cycling. The largest elastic recoil was observed in the non-reinforced/OC fenestrations (40%). A 10% elastic recoil was observed with LISF. Conclusion: In terms of mechanical behavior, the custom-made fenestration produced the highest results in terms of radial and branch pull-out strength. Both PMSG and LISF could be improved with the standardization of the fenestration creation protocol.

## 1. Introduction

Endovascular treatment with a fenestrated endograft (FE) is the first-line endovascular option for juxta and pararenal abdominal aortic aneurysm [1,2,3]. Current FEs include one to five fenestrations according to the number of target arteries [4]. These fenestrations are reinforced with the use of a nitinol-based wire ring surrounding the fenestrated zone on the fabric. The term “reinforced” is currently used to describe the shape memory of nitinol which helps to recover the geometry of the fenestration after it is released from the delivery system. The wire strand includes radiopaque markers made of gold, silver, or platinum for the purpose of fenestration localization [5].

The standard use of FE is, however, limited by significant manufacturing delays, anatomic standards, and the procedure’s cost. The latter is not acceptable for patients at high surgical risk in association with emergent situations. To fulfill these therapeutics dead-ends, physicians have developed different alternatives to create fenestration. Physician-modified stent-graft (PMSG) and laser in situ fenestration (LISF) have emerged to circumvent the restrictions raised by FE.

Both techniques lack standardization in the fenestration creation protocol [6]. Concerning PMSG, several surgical teams have reported various creation technical details to select stent-grafts, including the reinforcement material used, if used, the cutting material used, and its suture and stabilization with a bridging stent [7]. One may wonder if the generalization of the PMSG results is accurate, independent of the technical details. Moreover, PMSG can be used in many indications, mainly in aortic aneurysms and aortic dissection. Thus, for example, we can see technical differences in the aortic arch PMSG for aortic dissection compared to juxta-renal PMSG for aortic aneurysm [7,8,9].

LISF represents an alternative for complex endovascular aneurysm repair. The LISF concept is based on covering the aortic branches involved in repair with a standard endograft to further puncture and dilate the fabric to maintain branch perfusion. This technique was first described by Mc Williams et al. to preserve the left subclavian artery during a thoracic aneurysm endovascular repair [10]. ISF has been reported at both the level of the aortic arch and the abdominal level [11,12,13,14]. Two main methods can be considered to puncture the graft: (1) mechanical, mainly using a needle [15,16,17], and (2) physical, with laser catheters [18,19,20]. LISF has been suggested to be technically simpler and faster, especially in antegrade approaches [21].

The objective of this study was to compare the different fenestrations from a mechanical point of view.

## 2. Methods

### 2.1. Samples

In the frame of this work, the purpose was to assess the differences between the main fenestrated endograft commercially available: LISF and PMSG.

In total, five Zenith Cook fenestrations (Cook Medical, Bloomington, IN, USA) and five Anaconda fenestrations (Terumo Company, Inchinnan, UK) were included in this study. All fenestrations included were 8 mm custom-made fenestrations. Samples were recovered from the preoperative testing model for the Anaconda device and from the canceled procedure for the Zenith device. Laser ISF and PMSG were created on a Cook TX2 polyethylene terephthalate (PET) cover material (Cook Medical, Bloominghton, IN, USA).

Tests were performed with equipment that is commonly used for the evaluation of vascular prostheses according to IS0/FDIS 7198. However, they had to be specifically adapted because the tests performed were not standard as previously described [22,23,24]. Due to a lack of material, the five fenestrations were tested in terms of their radial strength, branch pull-out strength, and cyclic fatigue. Table 1 reports the number of fenestrations tested.

### 2.2. PMSG Fenestration Creation Protocol

All fenestrations were cut with a scalpel or an ophtalmologic cautery (OC). The fenestration was made by one operator (JC). Two reinforcements were considered: (1) a 35 mm Amplatz Goose Neck Snare Lasso (Medtronic, MN, USA) composed of a stainless steel wire enrolled by a coil; (2) Spartacore Hi-Torque wire 0.014’ (Abbott, Abbott Park, IL, USA) composed of 3 braided nitinol wires enrolled in a gold-plated tungsten coil. Only the distal extremity of both devices was used. These reinforcements were selected due to their main use in PMSG creation. Regarding the sutures, 3 different types of 5.0 non-resorbable sutures (polypropylene monofilament, polytetrafluoroethylene (PTFE) monofilament, and braided polyester) were elected to fix the reinforcement.

An average of 3 fenestrations (diameter 8 mm) were created in each stent-graft section between the stent segments, avoiding any contact with the stent-graft metallic parts. A calibrated 8 mm cylinder was used to define the size and to prevent variability issues. Two orthogonal measurements were taken across the fenestration to control the fenestration size. Next, depending on the fenestration category, the selected reinforcement was fixed using the selected suture. After preliminary deformation radial tests were performed, the best reinforcement-suture couple in terms of strength was identified, and an additional configuration was created (which increased the number of the suture pass (HNSP)).

### 2.3. ISF Endograft Material and Tested Samples

In total, five laser ISFs were created in the frame of this work. The fabric was punctured using a 0.9 mm Turbo Elite laser catheter connected to a CVX300 generator (Spectranetics, Colorado Springs, CO, USA) using a 50 mJ/mm^2^ fluence and a 50 Hertz rate. The fenestration was made by one operator (JC). Punctures were made perpendicularly to the fabric. The initial hole created was dilated in three steps using first a 2 mm non-compliant balloon (Abbott, Santa Clara, CA, USA) inflated with a 10 atm pressure, followed by a 4 mm cutting balloon (Boston Scientific, Natick, MA, USA) and an 8 mm non-compliant balloon inflated with a 10 atm pressure (Abbott, Santa Clara, CA, USA). The five fenestrations were tested in terms of their radial strength, branch pull-out strength, and cyclic fatigue.

### 2.4. Radial Strength Test

Mechanical radial strength tests were performed on fenestrations with an MTS Insight™ tensile testing machine (MTS system corporation, Eden Prairie, MN, USA). The test consists of inserting 2 half-cylinders (radius 3 mm) in an 8 mm diameter fenestration. One cylinder was kept fixed while the other one moved at the predefined strain rate (2 mm and 1 mm/min) up to 2 mm, which corresponds to a 25% diameter increase. To account for variability in positioning the half cylinders in the fenestration, a 0.1 N preload was applied to each sample. A total of 5 fenestrations characterized by a similar surface area could be tested for each device, and each test was repeated 3 times.

### 2.5. Branch Pull-Out Strength Test

In this approach, 2 testing configurations were considered in order to assess separately the effect of the fenestration size and the branch size on the assembling tightness. A calibrated 8 mm cylinder was introduced into each type of fenestration. The position of the endograft was maintained and fixed on a tensile testing machine by inserting 2 cylinders (characterized by the diameter of the graft) at both extremities. The pull-out cylinder was linked to the moving clamp and pulled at a speed of 20 mm/min over a 10 mm displacement distance. The pull-out force was measured and plotted vs. displacement. The goal was to compare the deformability of different fenestration designs.

The insertion depth of the cylinder was defined in order to reproduce the clinical practice, with approximately 1/3 of the stent-graft length on the aortic side and 2/3 on the target artery side.

### 2.6. Cyclic Fatigue Test

Cyclic fatigue tests were performed to study how the fenestration morphology was modified with time in each configuration. In the frame of this test, the stent graft was fixed on the fatigue testing bench frame while a 9 mm pin was introduced in the fenestration and linked to the moving axis. A 14 mm linear movement at 2 Hz frequency and over 7200 cycles (corresponding to 1 h cycling) was imposed on the linear axis. The branch was flexed over an approximately 30° angle range relative to the fenestration. These testing conditions were considered extreme loading conditions reproducing the relative movements which could occur in vivo between the stent and the main vessel under pulsatile heart loading. In the first approach, the cycling was conducted over 7200 cycles (representative of 1 h in vivo loading).

Fenestration morphologies were analyzed after cycling and were compared from a quantitative point of view: the surface area of the fenestrations was measured before and after fatigue to identify the potential deformations undergone by the reinforcement ring or locally in the textile. The analysis was performed on each device to bring out which design factors could have an influence on potential degradations.

### 2.7. Statistical Analysis

Quantitative variables were expressed as the mean (SD) for normal distribution and as medians (range) for non-normal distribution. A comparison of paired quantitative variables was performed with a paired t-test. A *p*-value < 0.05 was considered statistically significant.

## 3. Results

### 3.1. Radial Strength Test

**Figure 1:**
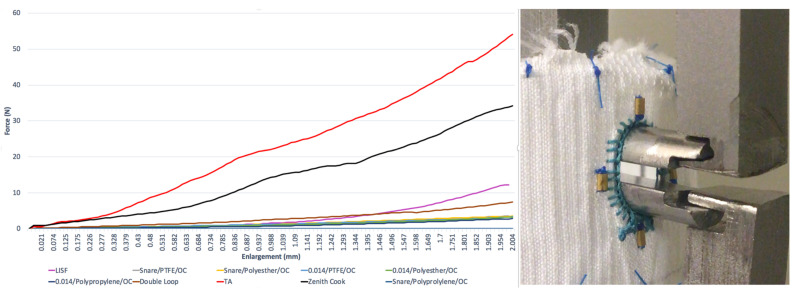
The load–displacement curves were obtained for each fenestration and the radial strength test model. LEGENDS: OC: ophtalmologic cautery; TA: Terumo Anaconda; LISF: laser in situ fenestration.

After 2 mm of vertical extension, the average radial strength was 54 ± 18 N for the Terumo Anaconda reinforced fenestration and 33.4 ± 6.9 N for the Zenith Cook fenestration. Figure 1.

After 2 mm, the LISF radial strength was 12.27 ± 6.08 N. Concerning PMSG, the highest radial strength was observed with a double loop of the snare (7.44 ± 2.9 N).

### 3.2. Branch Pull-Out Strength Test

**Figure 2:**
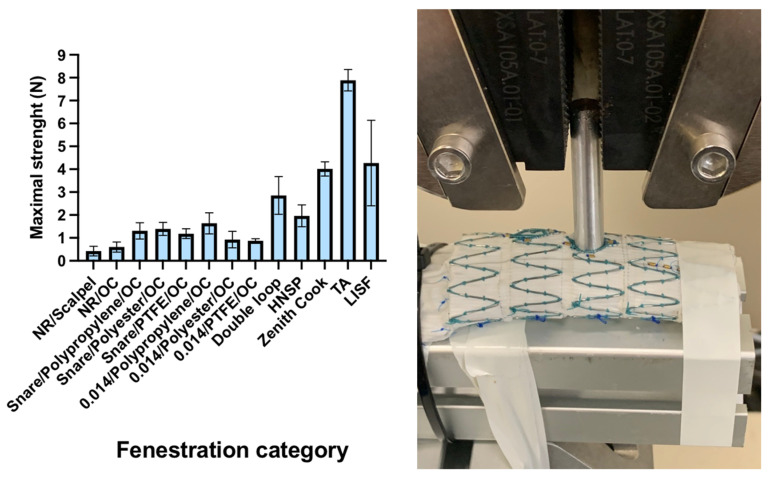
The results of the branch pull-out strength test for each type of fenestration and the pull-out strength test model. Legends: NR: non reinforced; OC: ophtalmologic cautery; HNSP: high number of suture pass; TA: Terumo Anaconda; LISF: laser in situ fenestration.

The extraction strength was higher with custom-made fenestrations, including both Zenith Cook and Anaconda fenestrations (9.5 ± 4.7 N and 4.49 ± 0.28 N) Figure 2. Double loop reinforced fenestrations were over two times radially stiffer than a simple reinforcement (2.7 N ± 0.8 vs. 1.1 N ± 0.3; *p* < 0.001). The extraction strength was increased in the HNSP configuration (1.8 N ± 0.4; *p* < 0.004). The comparison between LISF and double loop PMSG highlighted a higher force value with LISF (3.96 N ± 1.86 vs. 2.7 N ± 0.82; *p* = 0.018).

### 3.3. Cyclic Fatigue Test

The diameter of the fenestrations varied between 8 and 9 mm after cycling, while the initial diameter was 8 mm. As a 9 mm calibrated branch was inserted in the fenestration, we could consider that all fenestrations underwent a “textile elastic recoil” after cycling. An amplitude variation was observed between the samples. This phenomenon highlights the influence of the cyclical fatigue test on fenestration enlargement. Theoretically, the textile elastic recoil should have brought all fenestrations back to their initial diameter. The elastic recoil parameter was defined as the difference between the initial surface of the fenestration before fatigue and its dilated surface after the fatigue tests. The elastic recoil value was 100% if the size of the fenestration surface area remained unchanged after the test. However, in order to prevent the results from depending on the fenestration initial size variability in relation to the manual design, the calibrated branch diameter was considered in the calculation. For that purpose, the elastic recoil calculation was based on the comparison between the two following parameters: (1) the difference in terms of the surface area between the fenestration size before the test and the 9 mm caliper inserted in the fenestration over cycling; (2) the difference between the fenestration size before and after testing. 

We found a negative value because the fenestration area after cycling was lower than the calibrated branch area.

**Figure 3:**
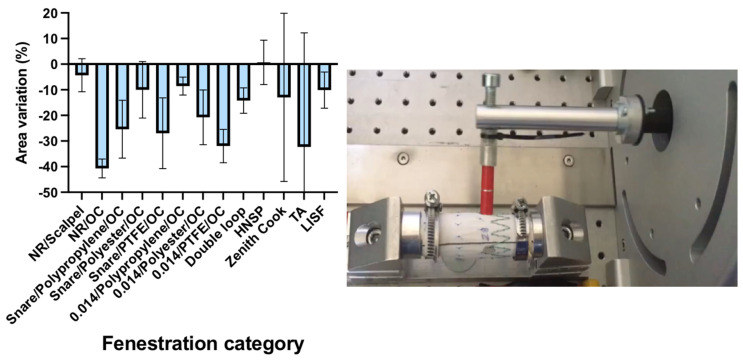
The fenestration size variations after a 7200 cycles fatigue testing and the fatigue bench test model. Legends: NR: N = non reinforced; OC: ophtalmologic cautery; HNSP: high number of suture pass; TA: Terumo Anaconda; LISF: laser in situ fenestration.

At the end of the test, the surface of the fenestration was increased for all fenestrations. Textile elastic recoil was incomplete for all fenestrations. The highest recoil was found with non-reinforced/OC fenestrations (40%), whereas the fenestrations created with the scalpel/scissor knives presented the lowest recoil value (4%), as the fenestrations were designed with an HNSP. Concerning the remainder of the samples, the elastic recoil varied between 11 and 35% Figure 3.

Terumo Anaconda fenestrations had the highest recoil compared with the Zenith Cook fenestration 30 vs. 12%. A 10% elastic recoil was observed with LISF.

At the end of the test, whatever samples were considered, no branch disconnection occurred.

## 4. Discussion

This study assessed the influence of the design of the fenestrations on the mechanical performance in terms of radial and pull-out strength and fatigue. The radial strength of custom-made fenestrations was four times higher than PMSG and LISF. As well as this, the required strength to extract the branch from the custom-made fenestration was higher. As a consequence, custom fenestrations could tighten in a larger way the balloon-expandable covered stent graft (CSG) inside the fenestration.

The interaction between CSG and the target artery plays a key role in mechanical strength and in sealing [25,26]. If resistance to the radial expansion of the fenestration on the CSG is not high enough, leakage can occur at the stent-fenestration junction. As a consequence, the insufficient strength of the connection could lead to type III endoleak, reinterventions, and the instability of the construction. Conversely, if the resistance to radial expansion is too important, the CSG can be locally compressed could generate a kink or stent fracture and early CSG thrombosis.

Commercially available FEs do not have the same design for fenestrations. Previous study results pointed out differences between the devices at various levels: (1) the radial strength of the fenestration; (2) the pull-out strength, and (3) the behavior under fatigue loading. We concluded that these differences could be related to variations in the construction of the devices [24]. We have observed a large range of values secondary to custom-made fenestration testing. One explanation could be the numerous variables between each device, as the variability of the number of sutures passed to reinforce the fenestration and the tension of the thread could vary between operators and influence the results.

When comparing PMSG and LISF, the results highlighted higher radial strength compared with double-loop reinforced fenestrations. In fact, we supposed that the reinforcement annihilated the textile elastic recoil phenomenon. The reinforcement could freeze the textile structure, whereas rearrangement occurred in the cases of LISF. This phenomenon was also observed in branch pullout strength tests.

After the analysis of the results of the fatigue tests, we observed that the stress-induced a stiffness variation in the fenestration for most of the designs. This observation was the most important with non-reinforced fenestration made by a knife or scissors. In fact, the textile elastic recoil was the lowest (4%), showing that the structure of the fenestration was continuously modified. Actually, after this modification of the textile, the yarns were free in the textile fabric construction and not fixed at their extremity, as could be observed with OC or LISF.

In addition, textile degradation could be found in the fabric located around the fenestration hole. It could be supposed that the cutting step may lead to textile tearing and be increased over the cyclic fatigue testing. When the OC was used, the melting zones tended to limit the movement of the yarns. Under fatigue, the fenestration was deformed but in an elastic way, as the yarns tended to come back to their initial position. Terumo Anaconda fenestration textile elastic recoil was the highest (35%). The design of the fenestration composed of nitinol wire without being fixed at its distal ends could explain the flexibility of the structure.

Limitations could be assigned to this work. First, in vivo conditions, such as fluid dynamics or blood conditions, were not reproduced. Secondly, the handmade design of devices included variability in the construction. Moreover, operator variability must be taken into account when the calibrated branch is introduced into the fenestration. We observed during LISF creation that the predilatation protocol with an 8 mm balloon did not create an 8 mm hole. In fact, the textile elastic recoil phenomenon reduced the diameter of the fenestration. This phenomenon has been already described in a previous study [17]. The textile tightens the interface between the pin and the fenestration in a larger way compared with PMSG. This could partially explain the higher value of strength during the radial and branch pull-out strength tests.

## 5. Conclusions

The mechanical behavior of the fenestrations was modified according to the design of the fenestration. In terms of mechanical performance, the custom-made fenestration performed the highest results in terms of the radial and branch pull-out strength. However, both PMSG and ISF techniques provide an interesting alternative. Both could be improved with the standardization of the fenestration creation protocol.

## Figures and Tables

**Table 1:** Number of fenestrations tested. LEGENDS: OC: Ophtalmologic cautery; TA: Terumo Anaconda; LISF: Laser in situ fenestration. NA: Not available.

Test	Radial Strength Test	Branch Pull-Out Strength Test	Cyclic Fatigue Test
LISF	5		
Zenith Cook	5		
TA	5		
HNSP	NA	5	5
Double Loop	5		
0.014/PTFE/OC	5		
0.014/Polyesther/OC	5		
0.014/Polypropylene/OC	5		
Snare/PTFE/OC	5		
Snare/Polyester/OC	5		
Snare/Polypropylene/OC	5		
NR/OC	NA	5	
NR/Scalpel	NA	5	

## Data Availability

No new data were created or analyzed in this study. Data sharing is not applicable to this article.
